# A point-of-care testing assay for clonorchiasis using a EuNPs-CsTR1 fluorescent probe-based immunoassay

**DOI:** 10.1371/journal.pntd.0012107

**Published:** 2024-04-24

**Authors:** Xiaoxiao Ma, Huiyuan Zhang, Yiming Fang, Jing Wang, Xingyang Wang, Chen Li, Xiaolei Liu, Mingyuan Liu, Bin Tang, Yi Liu

**Affiliations:** State Key Laboratory for Diagnosis and Treatment of Severe Zoonotic Infectious Diseases, Key Laboratory of Zoonosis Research, Ministry of Education, Institute of Zoonosis, College of Veterinary Medicine, Jilin University, Changchun, Jilin Province, China; Instituto de Salud Carlos III, SPAIN

## Abstract

*Clonorchis sinensis* is one of the most important fish-borne zoonotic parasitic worms in humans, and is distributed in several countries with more than 15 million people infected globally. However, the lack of a point-of-care testing (POCT) method is still the critical barrier to effectively prevent clonorchiasis. With the application of novel fluorescent nanomaterials, the development of on-site testing methods with high signal enhancement can provide a simple, precise and inexpensive tool for disease detection. In this study, Eu-(III) nanoparticles (EuNPs) were used as indicative probes, combined with *C*. *sinensis* tandem repeat sequence 1 (CSTR1) antigen to capture specific antibodies. Afterward, the complex binds to mouse anti-human IgG immobilized on the test line (T-line) producing a fluorescent signal under UV light. The EuNPs-fluorescent immunoassay (EuNPs-FIA) was successfully constructed, allowing sample detection within 10 min. It enabled both qualitative determination with the naked eye under UV light and quantitative detection by scanning the fluorescence intensity on the test line and control line (C-line). A total of 133 clinical human sera (74 negative, 59 clonorchiasis, confirmed by conventional Kato-Katz (KK) methods and PCR via testing fecal samples corresponding to each serum sample) were used in this study. For qualitative analysis, the cut-off value of fluorescence for positive serum was 31.57 by testing 74 known negative human samples. The assay had no cross-reaction with other 9 parasite-infected sera, and could recognize the mixed infection sera of *C*. *sinensis* and other parasites. The sensitivity and specificity of EuNPs-FIA were both 100% compared with KK smear method. Taking advantage of its high precision and user-friendly procedure, the established EuNPs-FIA provides a powerful tool for the diagnosis and epidemiological survey of clonorchiasis.

## Author summary

*Clonorchis sinensis* is one of the most important foodborne zoonotic parasites, affecting over 15 million people worldwide. However, the low-intensity infection and the lack of instant detection methods remain the main inhibitory factors for controlling this disease. In this study, the specific CSTR1 antigen was combined with EuNPs to construct a fluorescent probe to capture clonorchiasis-specific antibodies. Then, the complex binds to anti-human IgG immobilized on the test line, producing a fluorescent signal under UV light. The established EuNPs-FIA method enabled both qualitative determination with the naked eye and quantitative detection by scanning fluorescence probes intensity. The FIA has no cross-reaction with other 9 heterologous samples, and the sensitivity tests showed that the serum sample corresponding to 24 eggs per gram (EPG) in KK method can be still detected. Our work indicated that the established EuNPs-FIA method has broad application prospects in clinical and epidemiological studies and can aid in the surveillance and control of clonorchiasis.

## Introduction

Clonorchiasis, one of the most important foodborne parasitic zoonoses caused by *Clonorchis sinensis*, results in various complications in the liver and biliary systems, mainly cholelithiasis, cholangitis, and cholecystitis [1–2]. Approximately 15 million people are estimated to be infected with *C*. *sinensis*, with chief infections in China, South Korea, Northern Vietnam, and parts of Russia [3–4]. *C*. *sinensis* infection is an urgent public health problem due to the increasing risk of developing cholangiocarcinoma, and as a result, *C*. *sinensis* was classified as a Group 1 biological carcinogenic agent in 2009 [5]. Although pathogen monitoring programs implemented by the health sector are important for the prevention and control of clonorchiasis, it is still an uncontrolled disease in some areas, especially in resource-limited regions, due to the lack of accurate and sensitive detection methods.

Globally, the diagnosis of clonorchiasis has been primarily based on fecal examination using the Kato-Katz (KK) method, which is simplicity, low cost, and the ability to quantify infection intensity [6]. However, the KK method has a low sensitivity, especially for detection of low-intensity infection [1]. Although the sensitivity of KK method could be increased by increasing the number of tested samples, the labor-intensive and time-consuming drawbacks still limit its application [3–4]. In recent years, a variety of nucleic acid detection approaches, including recombinase polymerase amplification (RPA), polymerase chain reaction (PCR) and loop-mediated isothermal amplification (LAMP), have been used as detection methods [7–9]. Although such methods provide proven means for detection, the shortcomings include complexity, high cost, essential training and high-quality samples needed, limiting their use in resource-limited areas [10]. Serological methods are an effective means of rapid diagnosis of diseases at scale. In the diagnosis of clonorchiasis, excretory and secretory products (ESPs) are considered to be superior to crude extract, showing a sensitivity of 93.1% [11]. However, ESPs are difficult to produce in large quantities, in addition, are a mix of antigens, resulting in poor specificity [1,4]. Some recombinant proteins (7-kDa protein, 28-kDa cysteine protease, 26-kDa and 28-kDa glutathione S-transferase) have shown certain sensitivity and specificity in the serum diagnosis of clonorchiasis, but are not sufficient to replace crude extracts or ESPs [12–15]. Thus, further research is needed to determine more effective serological antigens.

Antigen with tandem repeat (TR) domains, defined here as two or more copies of an amino acid sequence, have been found in a variety of organisms ranging from prokaryotes to higher animals, and these proteins appear to be B-cell antigens [16]. Our recent studies demonstrated that the specific *C*. *sinensis* tandem repeat sequence 1 (CSTR1) antigen, serologically screened from *C*. *sinensis* expression library using pooled sera from clonorchiasis patients, indicate good sensitivity and specificity in detection. However, its use as a serum diagnostic antigen for establishing detection methods still needs further investigation.

The conventional immunochromatographic assay is convenient to detect pathogen infection within a few minutes, but the sensitivity needs to be improved [17]. Fortunately, with the application of nanomaterials, the detection signal of the test strip can be strongly enhanced, and the detection sensitivity can be greatly improved [18–19]. Eu (III) nanoparticles have the characteristics of a large Stokes shift (>150 nm), long fluorescence lifetime and low interference from background fluorescence, thereby leading to improved sensitivity [20–22]. EuNPs-based test strips have been used to detect a variety of pathogens, including bacteria, viruses and parasites [23–26]. However, the capacity of EuNPs-based fluorescent immunoassays for on-site detection of *C*. *sinensis* infection has not been explored.

Therefore, the objective of the present study was to develop a sensitive and intuitive EuNPs-CSTR1-based fluorescent immunoassay, and further evaluate its potential as a powerful tool for epidemiological surveillance of clonorchiasis.

## Materials and methods

### Ethics statement

All rabbits were handled strictly in accordance with the Animal Ethics Procedures and Guidelines of the People’s Republic of China, and the protocol was approved by the Institutional Animal Care and Use Committee of Jilin University (Protocol # 20170318).

All participants who were >18 years old were informed and enrolled in the research. This study was approved by the Ethical Committee of Jilin University, China (ethical clearance number # 2021703).

### Reagents and instruments

COOH-modified europium nanoparticles (EuNPs), were obtained from Thermo Fisher (USA); 1-ethyl-3-(3-dimethylaminopropyl) carbodiimide hydrochloride (EDC), N-hydroxysulfosuccinimide (NHS), were obtained from Tokyo Chemical Industry (TCI Shanghai, China); mouse anti-human IgG antibody, goat anti-rabbit IgG antibody, rabbit anti-goat IgG antibody, were obtained from Beijing Biolab Technology (Beijing, China); nitrocellulose filter membrane (CN140), sample pads (SB06, SB08, VL78, VL98 and RB45), absorbent pad, plastic backing, were obtained from Jinbiao Biotech (Shanghai, China); and bovine serum albumin (BSA), Tween-20, sucrose, were obtained from Solarbio (Beijing, China). The Time-resolved fluorescence (TRF) quantitative analyzer was obtained from Weice Biotech (Nanjing, China). The CSTR1 antigen, composed of four repeating units, was synthesized by Sangon Biotech (Shanghai) Co., Ltd. (Shanghai, China) at a concentration of 1 mg/mL.

### Parasites, sera and feces samples

*C*. *sinensis* metacercariae were obtained from naturally infected *Pseudorasbora parva* purchased from endemic areas in Changchun city, Jilin Province, China, and were orally administered to New Zealand white rabbits [27]. Adult worms were collected from the bile ducts of infected rabbits 5 weeks post-infection. Excretory-secretory products (ESPs) and crude antigens of adult worms were prepared as previously described [28]. A total of 151 human sera were used in this study, 59 serum samples were collected from patients with clonorchiasis (egg-positive as detected by the KK and PCR method), and healthy individuals without parasitic infections (n = 74), 9 mixed infection sera (2 cases of mixed infection sera of *C*. *sinensis* and *Schistosoma japonicum*, 3 cases of *C*. *sinensis* and *Trichinella spiralis*, 2 cases of *C*. *sinensis* and *Echinococcus granulosus* and 2 cases of *C*. *sinensis* and *Taenia solium*), and sera from patients with other parasite infections (n = 9) (*Schistosoma japonicum*, *Paragonimus westermani*, *Taenia solium*, *Trichuris trichura*, *Echinococcus granulosus*, *Ascaris lumbricoides*, *Ancylostoma duodenale*, *Trichinella spiralis* and *Toxoplasma gondii*) were obtained from Chinese Center for Disease Control, and stored in our institute. Besides, fecal samples corresponding to each serum sample were also collected, and the feces of the corresponding negative serum were tested three times by the KK and PCR methods to ensure that they were parasite free. The PCR primers CS1 (5′-CGAGGGTCGGCTTATAAAC-3′) and CS2 (5′-GGAAAGTTAAGCACCGACC-3′) were used to amplify the partial internal transcribed spacer 2 (ITS2) gene of *C*. *sinensis* [29]. The experiments in this study were approved by the Ethical Committee of the First Hospital of Jilin University, China (permit number: 2021703). All of the serum samples were collected from adults, and written informed consent was acquired from the adults before the samples were used.

### Preparation of polyclonal antibodies against CSTR1 antigen

Healthy male New Zealand white rabbits weighing 2-2.5kg were selected to prepare polyclonal antibodies. For the first immunization, 800 μg of CSTR1 antigen emulsified in Freund’s complete adjuvant was subcutaneously injected into the back of rabbits through multi-site injection. During the next three immunizations, 400 μg of CSTR1 antigen emulsified in Freund’s incomplete adjuvant was injected at a two-week interval. After one week of the last immunization, the serum was collected from the rabbits’ common carotid artery. The antibody was purified by protein G sepharose affinity column and stored at -20°C. The western blotting was also performed, and the ESPs and crude antigens of adult worms were incubated with anti-CSTR1 polyclonal antibody separately to verify the presence of CSTR1 in its native state. All experiments were approved by the Ethical Committee of Jilin University, China (ethical clearance number: 2021530).

### Preparation of the fluorescent probe

EuNPs were conjugated with CSTR1 antigens (EuNPs-CSTR1) as previously described [13]. First, 10 μL of EuNPs were centrifuged at 15 000×g for 20 min to remove glycerol and phosphate, and then 10 μL of EDC (1 mg/mL), 10 μL of NHS (1 mg/mL), and 100 μL of MES (0.05 M) were mixed with EuNPs evenly for 45 min (dark conditions, room temperature) to completely activate the EuNPs. Second, the unbound EDC and NHS were removed at 15 000 × g for 20 min, and then 20 μg of CSTR1 antigen was mixed into the composites and incubated for 3 h (dark conditions, room temperature). Third, 200 μL of 5% BSA was added to the mixtures to block the unreacted active sites (4°C, 12 h). Finally, the EuNP-CSTR1 fluorescence probe was resuspended in 200 μL of preservation buffer (0.05 M PBS containing 1% BSA and 1% ProClin) and stored at 4°C under dark conditions. The EuNPs-goat anti-rabbit IgG fluorescent probe was also prepared by the same procedure described above.

### Preparation of the fluorescent immunoassay

**Fig 1:**
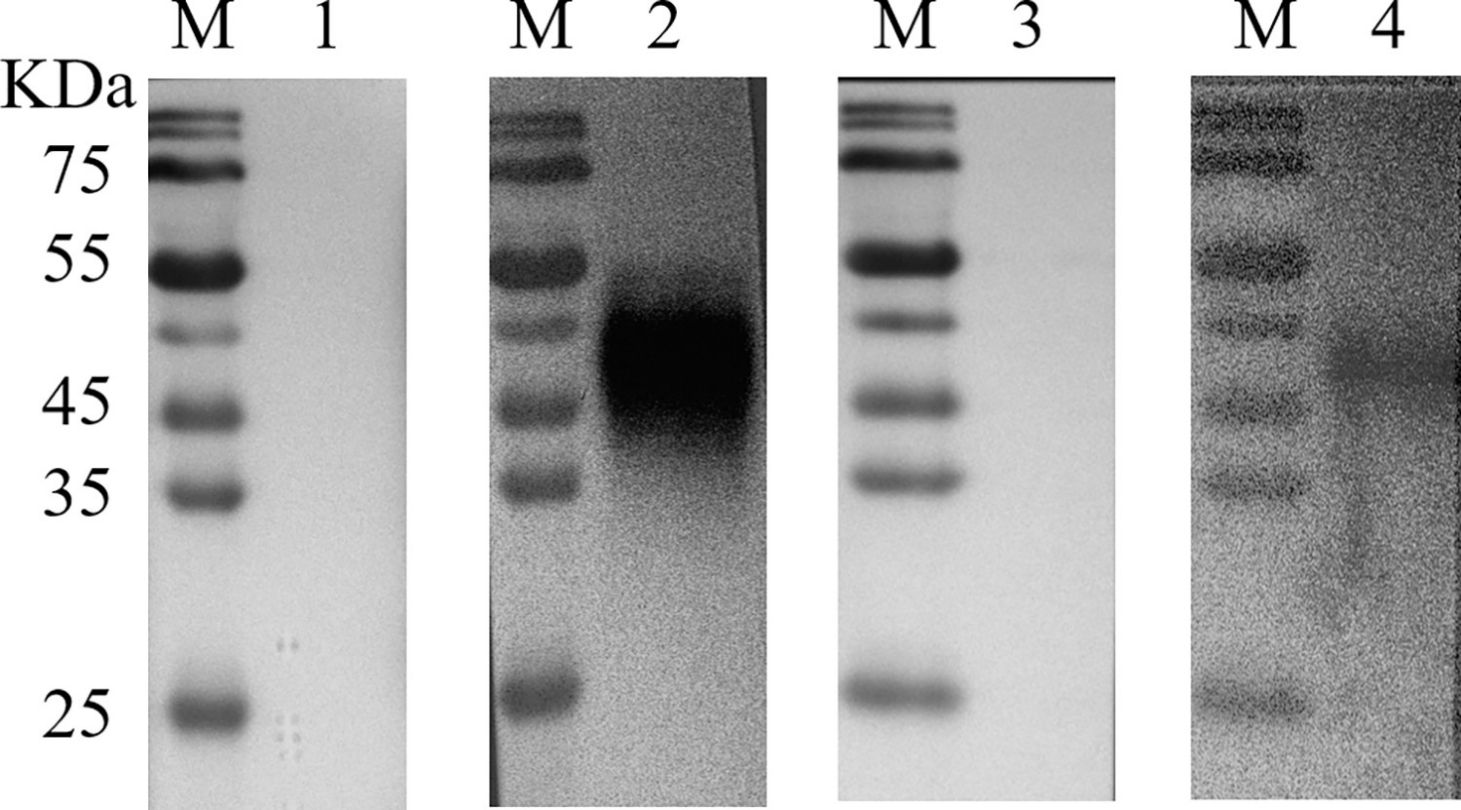
The schematic diagram and reading of the EuNPs-FIA. (A) The composition of immunochromatographic strips and the process of immunochromatographic strips. (B) The image of positive and negative serum samples detected by FIA under UV lamp. (C) FIA fluorescence curves of positive and negative serum sample analyzed by TRF reader.

The fluorescent immunoassay strip consists of sample pad, nitrocellulose filter membrane (NC membrane), absorbent pad and plastic backing. Two fluorescent probes were designed, using EuNPs-conjugated CSTR1 antigen as the capture probe to capture anti-*C*. *sinensis* antibodies, and the EuNPs-conjugated goat anti-rabbit IgG antibody as indicator probe. Mouse anti-human IgG (1 mg/mL) and rabbit anti-goat IgG (1 mg/mL) were applied to the NC membrane as the test line (T-line) and control line (C-line) by an XYZ3060 dispenser at a rate of 0.5 μL/cm, respectively. Finally, the sample pad, NC membrane and absorbent paper were pasted on the plastic backing in order and then assembled into strips (3.5 mm wide) (Fig 1A).

During testing, 94.25 μL running buffer (10% sucrose, 1% BSA, 1.6% Tween-20), 5 μL serum sample, 0.5 μL EuNPs-CSTR1 and 0.25 μL EuNPs-goat anti-rabbit IgG were mixed into a tube. Then, the mixture was added to the FIA strip and placed into a 37°C incubator for 10 min. For result interpretation, the positive sample could have the fluorescent signals on both the T-line and C-line under a UV lamp at a 365 nm wavelength; for the negative sample, only the C-line has a fluorescent signal (Fig 1B). Additionally, the fluorescence intensity signal was also analyzed by a TRF reader.

### The mechanism and reading of the FIA

The principle of the FIA can be considered a classical indirect detection method. There are four parts (from left to right): a sample pad for filtering liquid samples; an NC membrane for loading mouse anti-human IgG and goat anti-rabbit IgG; an absorption pad for liquid absorption; and a PVC plate for supporting these four components. When the sample solution is added to the sample pad, it will migrate toward the conjugate pad under the action of a siphon (Fig 1A). In addition to qualitative visual judgment, the strip results can also be accurately read by the TRF fluorescence quantitative analyzer for quantitative analysis (Fig 1C).

### Optimization of key parameters

To achieve a perfect release of fluorescent particles, several relevant reaction conditions were optimized. Firstly, the materials of the sample pads SB06, SB08, VL78, VL98 and RB45 were evaluated for suitability of the FIA. Secondly, the conjunct concentration between fluorescent particles and CSTR1 antigen was tested at ratios of 10:1, 20:1, 30:1 and 40:1 on the FIA. Finally, the dilution ratio of serum was optimized at 1:5, 1:10, 1:20, 1:30, and 1:40. For all optimization processes, once a condition is selected as the optimum, it will remain fixed when exploring other conditions. For each optimization, each sample was tested three times and the visual results were observed with a 365 nm UV lamp, and the fluorescence values were quantitatively analyzed by the TRF reader.

### The cut-off value for the FIA

A total of 74 negative sera, confirmed by the PCR and stool KK method, were detected by FIA, and the fluorescence values were analyzed by the TRF reader in triplicate. The cut-off value was calculated by means ± 3 SD of the T-line fluorescence values. Samples with a T-value lower than the cut-off were considered negative, and samples higher than or equal to the T-value were judged as positive.

### Sensitivity and specificity of the FIA

Serum samples were divided into different categories based on the infection intensity observed by KK in the corresponding stool samples (EPG: 0, 24, 48, 72, 96, 100, 144, 168, 192, 288, 384, 480), and each serum sample was detected by FIA to evaluate its sensitivity. Each of the reactions was performed in triplicate according to the predetermined optimal conditions. The specificity of the FIA was evaluated by cross-reaction tests using 9 other parasites (see details above), and all of these sera were tested in triplicate under the optimal conditions.

### Applicability of the FIA

The diagnostic effectiveness of the EuNPs-CSTR1-based FIA was evaluated using 142 human sera and corresponding stool samples, which were randomly selected from residents of Fuyu County (Jilin Province, China), where clonorchiasis is endemic. Each serum sample was analyzed by EuNPs-CSTR1 FIA in triplicate under the optimal conditions. The 59 clonorchiasis positive fecal sample was divided into two subsamples: (1) one part was tested with the KK technique, and (2) one part was tested with the PCR method. DNA from human stool samples was extracted using the QIAamp Tissue Kit Spin Columns (Qiagen, Hilden, Germany) following the manufacturer’s protocols. To analyze the performance of the FIA for detection of clonorchiasis, 59 positive and 74 negative serum samples were analyzed by FIA, and the receiver operating characteristic (ROC) curve was drawn based on the fluorescent intensity of FIA.

### Statistical analysis

The fluorescence intensity of the T-line of EuNPs-FIA was analyzed by a TRF, and the results were expressed using GraphPad Prism8. The kappa (κ) coefficient was employed by the statistical package SPSS.

## Results

### Validation of CSTR1 antigens

**Fig 2:**
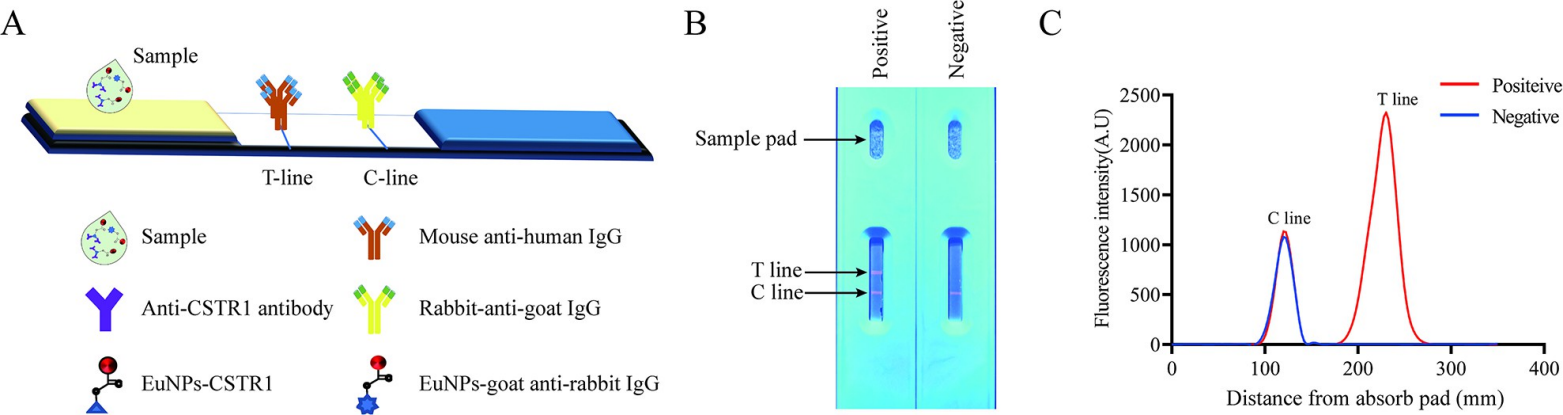
Western blotting of CSTR1 with *C*. *sinensis* ESPs and crude antigens. M: protein marker; 1: ESPs antigens incubated with negative serum; 2: ESPs antigens incubated with anti-CSTR1 polyclonal antibody; 3: Crude antigens incubated with negative serum; 4: Crude antigens incubated with anti-CSTR1 polyclonal antibody.

The CSTR1 antigen, serologically screened from the *C*. *sinensis* expression library using pooled sera from clonorchiasis patients, is composed of multiple repeating units (GGKAPPPESAGGKAPPPESA). Western blotting results showed that CSTR1 antigen can be recognized by the anti-CSTR1 polyclonal antibody both in ESPs and crude antigens of adult worms, and the amount of CSTR1 in ESPs is much higher than in crude antigens (Fig 2).

### Parameter optimization of the FIA

**Fig 3:**
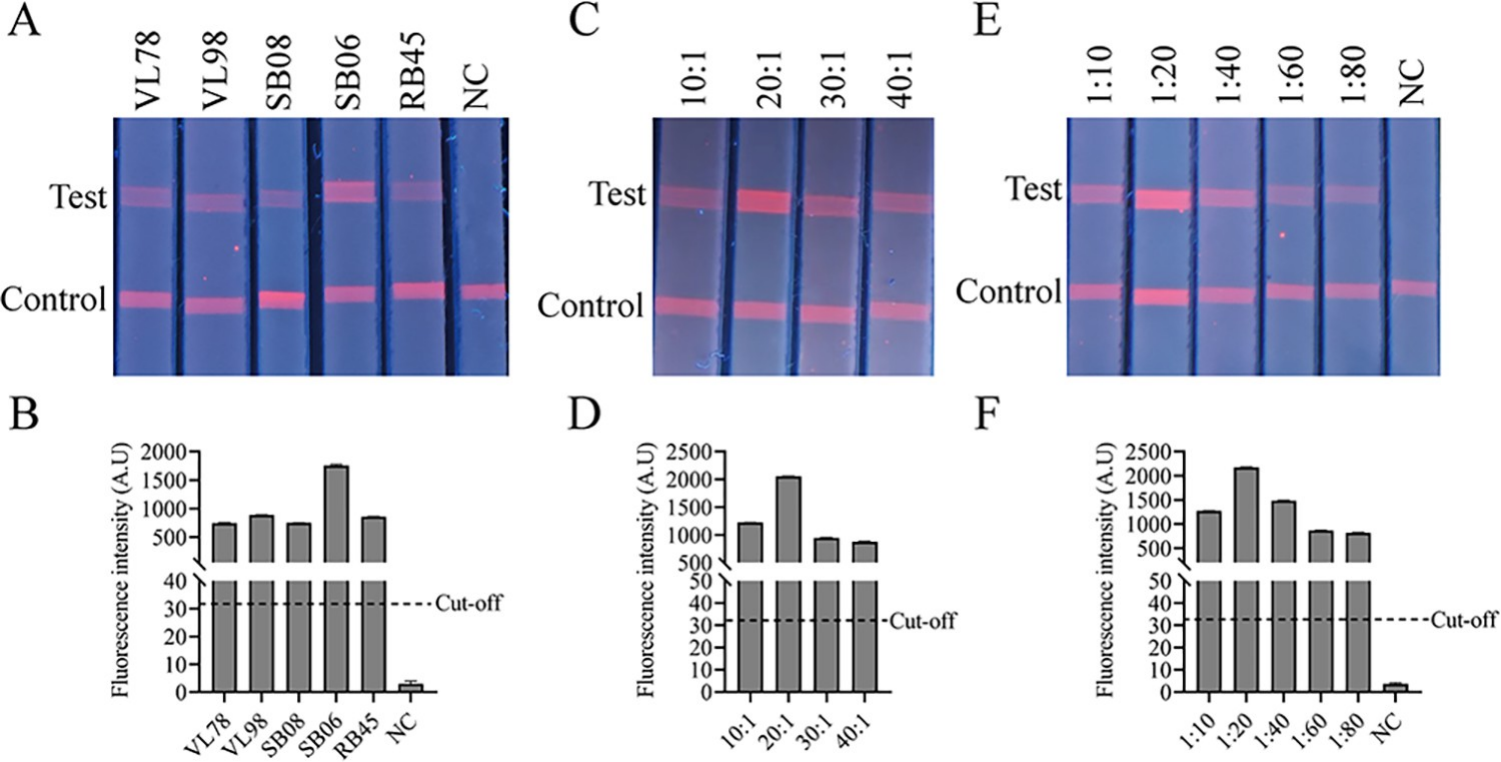
Optimization of the FIA. (A) The rate of CSTR1 antigen conjugated with fluorescent particles. (C) The materials of sample pads: VL78, VL98, SB08, SB06 and RB45. (E) The dilution ratio of serum. (B, D, F) FIA was quantitatively analyzed by the TRF reader.

The FIA could distinguish negative and positive serum samples clearly by rapid visual detection or accelerated machine reading, and the entire detection experiment could be completed within 10 minutes. Several reaction conditions were tested in the FIA to obtain the best detection results. Five sample pads were selected to determine their performance, and it was determined that the SB06 sample pad produced the best result (Fig 3A and 3B). The concentration of the conjugate EuNPs-CSTR1 was tested, and the use of 1 μg fluorescent particles at a conjugated ratio of 20:1 yielded the best result (Fig 3C and 3D). In addition, the dilution ratio of serum, 1:20, was optimized as the best condition, which brought out the strongest fluorescent signal on the T line (Fig 3E and 3F). Then, the SB06 sample pad, the fluorescent particles conjugated ratio of 20:1 and the serum dilution ratio of 1:20 were selected as the FIA reaction conditions.

### Cut-off value of the FIA

74 serum samples from parasite-free humans were tested by the optimized FIA. The fluorescence intensity of each sample was quantified via a TRF reader in triplicate. The means and standard deviation of these sera were 10.51 and 7.02, respectively. Based on the means ± 3SD value, the cut-off value for the EuNPs-CSTR1 FIA was 31.57.

### Sensitivity and specificity analysis of FIA

**Fig 4:**
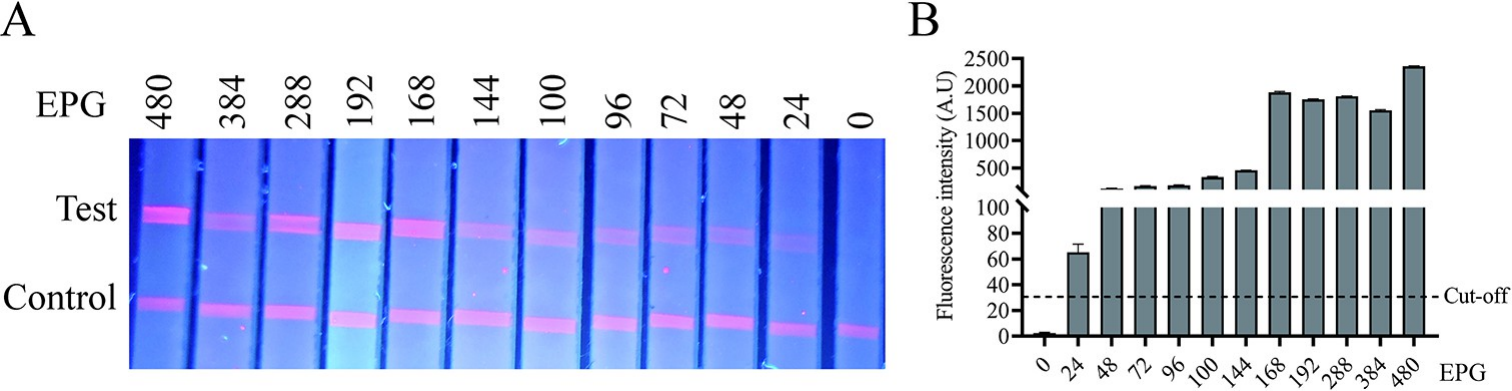
Sensitivity analysis of the FIA. (A) The FIA detected sera with different infection grades based on EPG (from 0 to 480, conformed by KK method), and the visual results of each serum under UV light. (B) FIA was analyzed by the TRF reader.

The sensitivity of the FIA was determined by testing different infection intensity serum samples, which were confirmed by KK method in the corresponding stool samples (EPG: 0, 24, 48, 72, 96, 100, 144, 168, 192, 288, 384, 480). The results indicate that the serum sample corresponding to 24 EPG in fecal examination can still be detected, with a fluorescence value of 78, which showed high sensitivity (Fig 4A and 4B).

**Fig 5:**
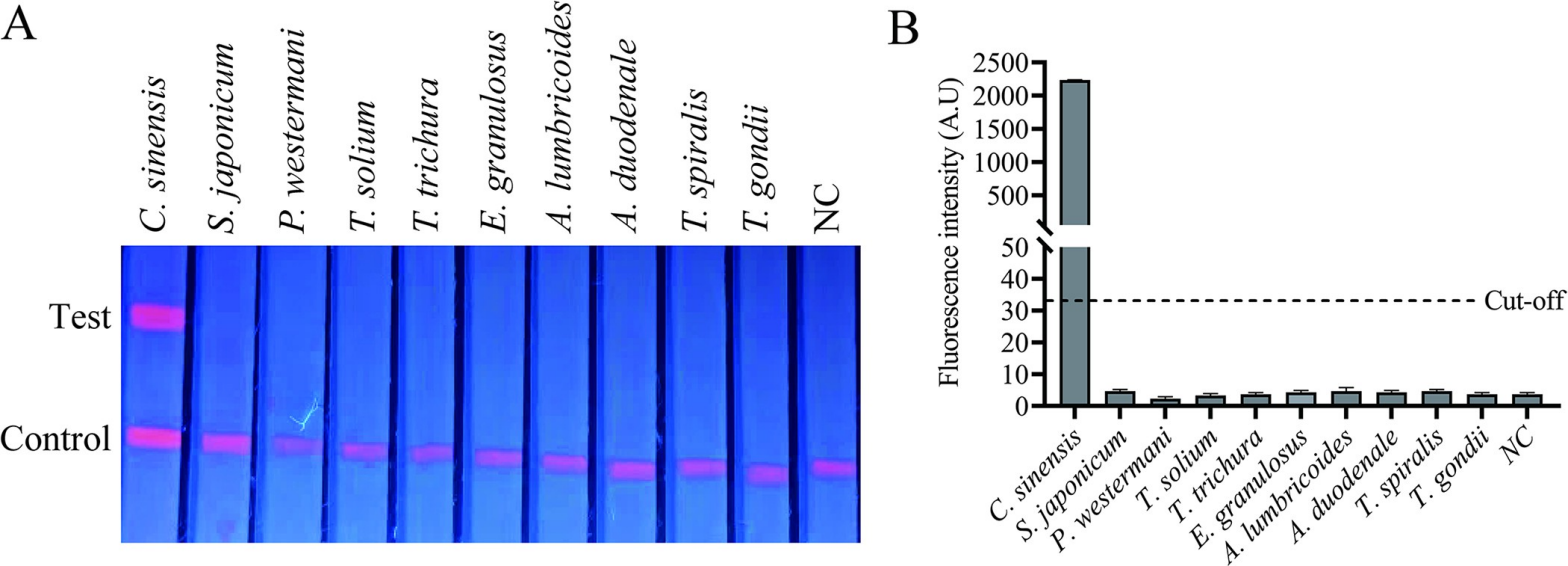
Specificity analysis of the FIA. (A) serum samples from human infected with different parasites were detected by EuNPs-FIA. (B) Fluorescence values of FIA analyzed by TRF reader.

To evaluate the specificity of the FIA, the FIA was performed under the optimal conditions by using 9 sera from other parasites, namely, *S*. *japonicum*, *P*. *westermani*, *T*. *solium*, *T*. *trichura*, *E*. *granulosus*, *A*. *lumbricoides*, *A*. *duodenale*, *T*. *spiralis* and *T*. *gondii*. There was no cross-reaction with any of the heterologous control samples, suggesting high specificity of the FIA in distinguishing *C*. *sinensis* from other pathogens (Fig 5A and 5B).

### Clinical application of FIA

**Fig 6:**
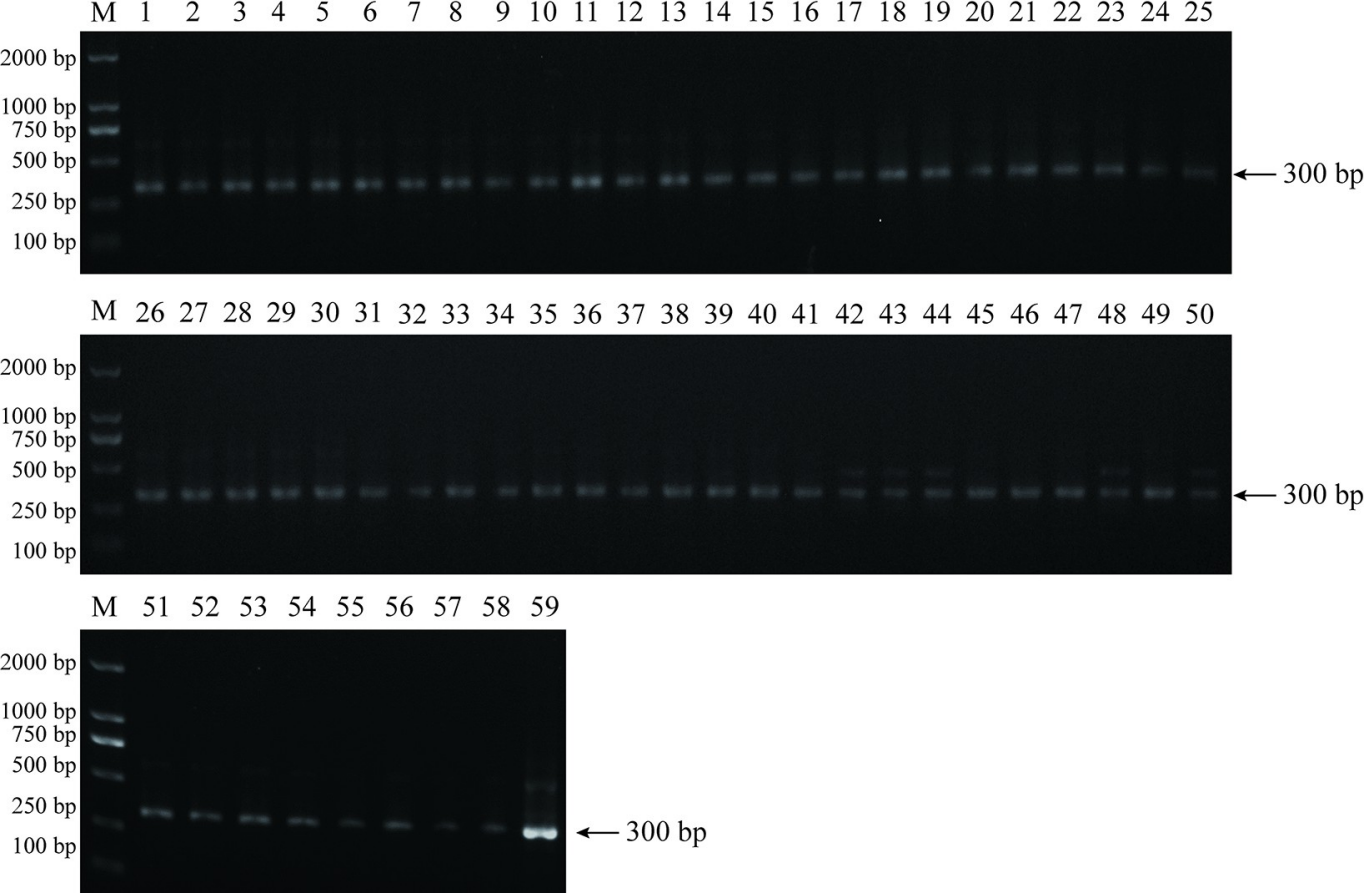
PCR amplification of fecal samples from 59 clonorchiasis. PCR amplification of *C*. *sinensis* ITS-2 gene was performed on 59 clonorchiasis fecal samples, with a target gene size of 300 bp.

**Fig 7:**
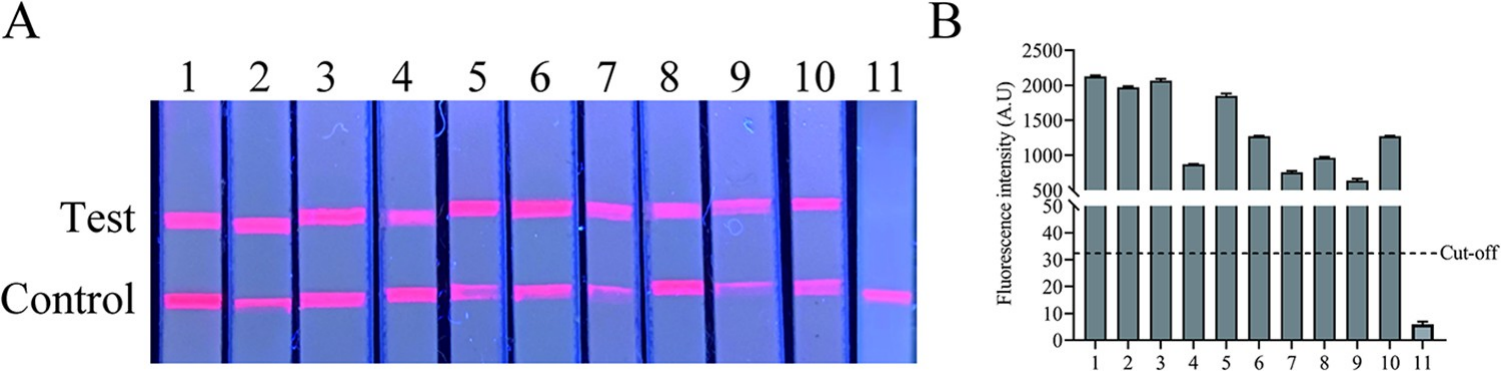
Application of FIA to mixed infection serum samples. (A) The FIA detected sera with mixed infection samples, and the visual results of each serum under UV light. 1: The positive serum of clonorchiasis; 2-3: Mixed infection sera of *C*. *sinensis* and *S*. *japonicum*; 4-6: Mixed infection sera of *C*. *sinensis* and *T*. *spiralis*; 7-8: Mixed infection sera of *C*. *sinensis* and *E*. *granulosus*; 9-10: Mixed infection sera of *C*. *sinensis* and *T*. *solium*; 11: Negative control. (B) Fluorescence values of FIA analyzed by TRF reader.

The performance of clinical application of the FIA was evaluated using 59 clonorchiasis, 74 negative and 9 mixed infection sera and their corresponding fecal samples. The 59 clonorchiasis positive sera were divided into different infection grades according to the corresponding stool KK test results (Table 1), and the FIA assay could detect different intensities of infection in the range of 24 to 480 EPG. After PCR amplification and sequencing, the fecal samples corresponding to 59 positive serum samples were confirmed as positive for *C*. *sinensis* (Fig 6). The 9 mixed infection sera of *C*. *sinensis* and other parasites could also be diagnosed by the FIA assay (Fig 7A and 7B).

**Table 1:** Comparison of the detection results between Kato-Katz and EuNPs-FIA.

Case code	Kato-Katz (EPG)	LFA (T-value)	Case code	Kato-Katz (EPG)	LFA (T-value)
A 0616	24	56	D 1756	96	677
A 0645	24	45	D 2007	96	682
A 0620	24	93	E 0198	100	688
A 1038	24	87	E 0068	100	616
A 7061	24	82	E 0472	100	528
A 0322	24	66	F 9410	144	588
A 1074	24	85	F 7830	144	569
A 1124	24	59	G 10119	168	639
A 0801	24	81	G 8892	168	656
A 0645	24	95	H 8819	192	676
A 1107	24	96	H 6960	192	755
B 5500	48	98	I 5325	240	757
B 5346	48	98	I 5489	240	766
B 5466	48	116	I 5531	240	1266
B 5342	48	121	I 5354	240	1369
B 6057	48	121	J 6782	288	1256
B 5881	48	151	J 0511	288	1363
B 5901	48	163	J 0063	288	1356
B 5828	48	177	K 0210	336	1231
C 8404	72	182	K 0241	336	1123
C 5623	72	233	K 0218	336	1445
C 5479	72	196	K 9269	336	1624
C 5459	72	305	L 10150	384	1587
C 5840	72	318	L 9056	384	1735
C 5819	72	471	L 8452	384	1828
C 5456	72	352	M 0041	432	1866
D 5455	96	464	M 9420	432	1774
D 5457	96	355	N 1280	480	2144
D 5373	96	474	N 9930	480	2205
D 6253	96	363			

**Fig 8:**
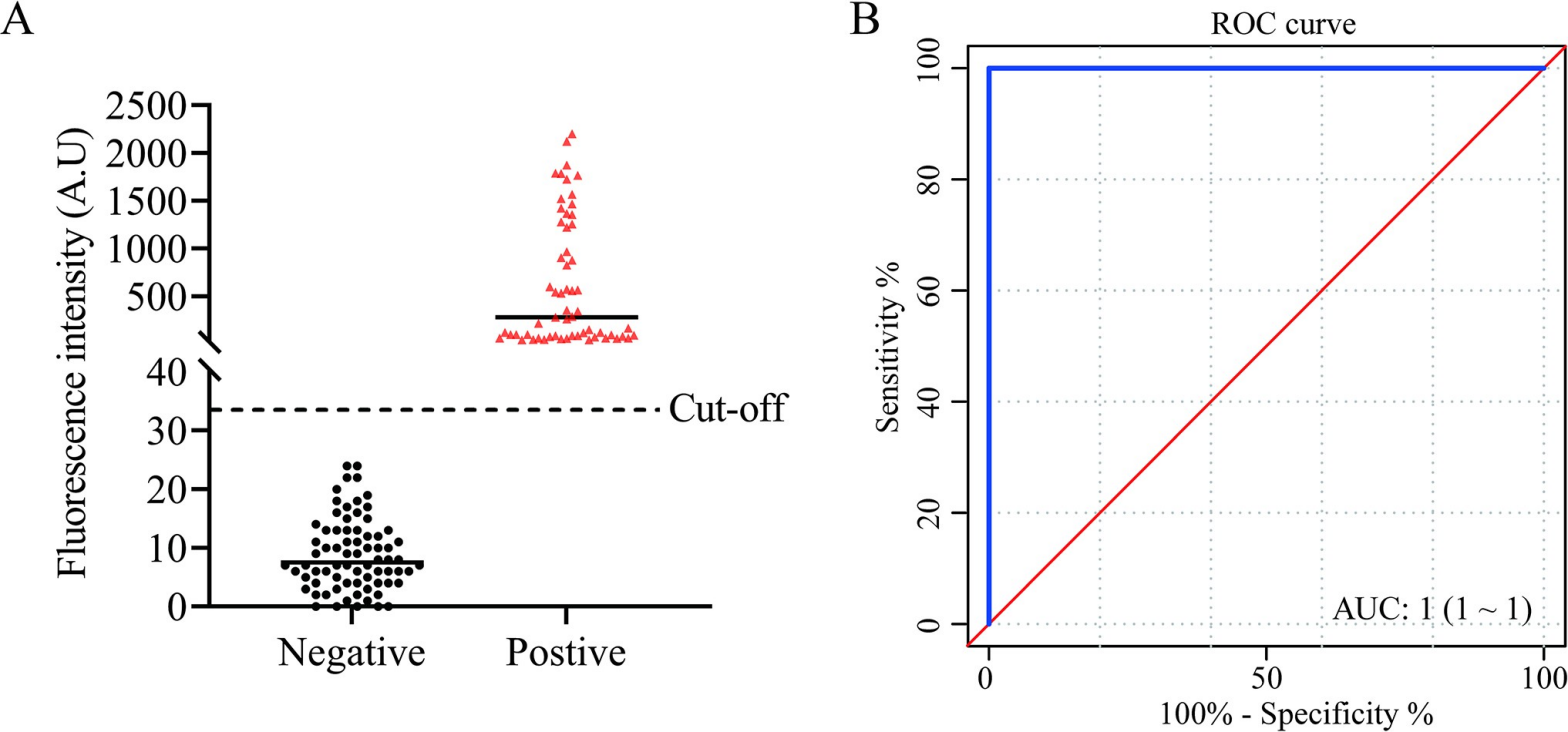
Application of FIA. (A) Distribution of negative and positive serum samples detected by FIA. (B) ROC analysis of serum samples based on the ratio values of the assay, and the integrated area under the ROC curve was 1.

The analysis of the ROC curve results of 59 positive and 74 negative serum samples showed that the area under the curve was 1.000 (positive coincidence rate: 100%, negative coincidence rate: 100%) with substantial kappa value (κ = 1), indicating that the assay can be used to accurately detect anti-*C*. *sinensis* IgG antibodies in human serum samples (Fig 8A and 8B and Table 2).

**Table 2:** Diagnostic performance of EuNPs-FIA compared with Kato-Katz method.

Classified by Kato-Katz method (as the gold standard)	Classified by EuNPs-FIA		Total
	T_+_^a^	T-^b^	
D_+_^c^	59	0	59
D-^d^	0	74	74
Total	59	74	133
*Se* ^e^	1.000		
*Sp* ^e^	1.000		
κ^f^	1.000		

^a^ EuNPs-FIA positive samples.

^b^ EuNPs-FIA negative samples.

^c^ Kato-Katz positive samples.

^d^ Kato-Katz negative samples.

^e^ The calculated sensitivity (*Se*) and specificity (*Sp*) of the EuNPs-FIA method.

^f^ The interpreted κ value of the EuNPs-FIA method

## Discussion

*Clonorchis sinensis* is one of the most important foodborne zoonotic parasites, affecting over 15 million people worldwide [1]. The low-intensity infection of *C*. *sinensis* remains a serious obstacle and is also the main inhibitory factor for the prevention and control of clonorchiasis [4]. Therefore, sensitive detection of this disease has important significance, especially in rapid testing. Several conventional detection techniques have been established, such as PCR, LAMP and ELISA, but due to the need for specific equipment and time-consuming procedures, these methods are not suitable for field applications [30–33]. Nevertheless, traditional fecal examination is considered the gold standard, although it has some drawbacks, such as false negatives and egg morphology similar to other flukes [1,4].

Thus, in the present study, we provided a novel and accurate EuNPs-CSTR1 FIA assay that allows for the point-of-care detection of clonorchiasis within 10 min. This method is easy to operate, environmentally friendly, and has no professional requirements. More importantly, it has high specificity and sensitivity, providing a powerful tool for the real-time diagnosis of clonorchiasis in resource-limited areas. It is noteworthy that there are no previous reports of an EuNPs-based FIA assay for clonorchiasis detection from human serum samples. The novel *C*. *sinensis*-specific CSTR1 antigen, serologically identified from the *C*. *sinensis* expression library using pooled sera from clonorchiasis patients, was selected as the diagnostic antigen for the first time.

Antigens containing TRs often serve as targets of B-cell responses [11]. In addition, TR antigens have been found in a variety of parasites, including *Fasciola hepatica*, *Leishmania donovani*, *Plasmodium falciparum* and *Trypanosoma cruzi* [16,34–36]. Regarding *C*. *sinensis*, some currently validated antigens are also TR proteins, such as Cs31 [37], CsPRA [38], CsGRP [39] and Cs1 [11], and they are genus-or species-specific [40]. Western blotting was used to identify whether CSTR1 is a component of *C*. *sinensis* ESPs or crude antigens, and the results showed that CSTR1 was recognized by the anti-CSTR1 polyclonal antibody (CSTR1-pAb). CSTR1-pAb could recognize the native CSTR1 protein of 45 kDa in ESPs and crude antigens of *C*. *sinensis* adult worms. These results indicate that CSTR1 is a component of both the ESPs and crude antigens of *C*. *sinensis*.

In addition to the specific antigen, the application of nanomaterials plays important auxiliary roles in enhancing the detection signal and greatly improves the detection sensitivity of the test strip [17,41]. Although a colloidal gold immunochromatographic test has been established for the detection of opisthorchiasis and clonorchiasis [42], a fluorescent probe-based FIA as a new method was introduced in the detection of *C*. *sinensis* infection here. Compared with the colloidal gold ICT, the EuNPs-based FIA could not only distinguish clonorchiasis-positive serum pointedly, and the result can rapidly be judged by naked eye observation and more precisely by a fluorescence reader. The feasibility of the established method has been verified, and there is no cross-reaction with 9 heterologous control samples, indicating that the FIA method has high specificity in distinguishing *C*. *sinensis* from other parasites. The sensitivity test results indicate that the low-intensity serum sample corresponding to 24 EPG in a KK microscopy slide can still be detected. The FIA method has shown excellent performance in clinical applications, detecting different intensities of infected sera in the range of 24 to 480 EPG, and the detection results were 100% sensitive and specific consistent with the KK smear assay.

China has a large population base, and more patients with clonorchiasis, with a relatively scattered distribution [1,3]. Scale based screening and immediate precision medical treatment are of great significance for the prevention and control of the pathogen at its source, especially in remote areas with limited resources [4]. Therefore, newly established POCT methods should comply with the “ASSURE” standard, which includes affordability, sensitivity, specificity, user friendliness, speed and repeatability, and equipment free. The EuNPs-FIA described in this study can be used for serological screening of large-scale populations, as it is easy to operate and requires low equipment requirements, which facilitate its application in remote areas. In addition, the fast and convenient interpretation greatly reduces the professional requirements for operators. However, this work still has certain limitations: firstly, the number of cross-reaction sera from other parasites is insufficient, which cannot fully verify its detection performance; secondly, the positive serum we currently use is a single infection of *C*. *sinensis*, lacking detection performance data for mixed infection cases; thirdly, although the detection process requires simple equipment, the demand for UV lamp to some extent limited its application. Last but not least, cross-reacting antibodies from previous infections may interfere with serological tests, leading to false positives, which is an inevitable issue in serological testing. Anyway, based on current experimental results, the EuNPs-FIA and KK method exhibit the same sensitivity and specificity. In the actual large-scale screening of clonorchiasis, it can be considered to first use EuNPs-FIA for rapid initial screening, and then use KK method to confirm positive results, which will greatly reduce the workload.

In summary, the novel EuNPs-based FIA assay allows for the point-of-care detection of clonorchiasis within 10 min. The assay has high specificity and sensitivity and is convenient to perform and assess, allowing it to be more applicable to field laboratories. In addition, the results can not only be qualitatively judged visually but can also be accurately read for quantitative analysis. This technique has broad application prospects in clinical and epidemiological studies and can aid in the surveillance and control of clonorchiasis.
